# Prediction of relapse in stage I testicular germ cell tumor patients on surveillance: investigation of biomarkers

**DOI:** 10.1186/s12885-020-07220-6

**Published:** 2020-08-05

**Authors:** João Lobo, Ad J. M. Gillis, Annette van den Berg, Leendert H. J. Looijenga

**Affiliations:** 1 Princess Máxima Center for Pediatric Oncology, Heidelberglaan 25, 3584 CS Utrecht, The Netherlands; 2 Department of Pathology, Portuguese Oncology Institute of Porto (IPOP), R. Dr. António Bernardino de Almeida, 4200-072 Porto, Portugal; 3 Cancer Biology and Epigenetics Group, IPO Porto Research Center (GEBC CI-IPOP), Portuguese Oncology Institute of Porto (IPO Porto) & Porto Comprehensive Cancer Center (P.CCC), R. Dr. António Bernardino de Almeida, 4200-072 Porto, Portugal; 4 Department of Pathology and Molecular Immunology, Institute of Biomedical Sciences Abel Salazar, University of Porto (ICBAS-UP), Rua Jorge Viterbo Ferreira 228, 4050-513 Porto, Portugal; 5 Department of Pathology, Lab. for Exp. Patho-Oncology (LEPO), Erasmus MC-University Medical Center Rotterdam, Cancer Institute, Doctor Molewaterplein 40, 3015 GD Rotterdam, the Netherlands

**Keywords:** Biomarkers, Immunohistochemistry, Relapse, Testicular germ cell tumors, Vascular invasion

## Abstract

**Background:**

Better biomarkers for assessing risk of relapse in stage I testicular germ cell tumor patients are needed, to complement classical histopathological variables. We aimed to assess the prognostic value of previously suggested biomarkers, related to proliferation (MIB-1 and TEX19) and to immune microenvironment (CXCL12, CXCR4, beta-catenin and MECA-79) in a surveillance cohort of stage I testicular germ cell tumor patients.

**Methods:**

A total of 70 patients were included. Survival analyses were performed, including Cox regression models.

**Results:**

Patients with vascular invasion and elevated human chorionic gonadotropin levels showed significantly poorer relapse-free survival in multivariable analysis (hazard ratio = 2.820, 95% confidence interval 1.257–6.328; hazard ratio = 3.025, 95% confidence interval 1.345–6.808). Patients with no vascular invasion but with MIB-1 staining in > 50% tumor cells showed significantly shorter relapse-free survival (*p* = 0.042). TEX19 nuclear immunoexpression was confirmed in spermatogonial cells, and weak cytoplasmic immunoexpression was depicted in 15/70 tumors, not significantly impacting survival. CXCL12 immunoexpression in tumor cells did not associate with relapse, but non-seminoma patients exhibiting vascular invasion and CXCL12-positive stromal/inflammatory cells showed significantly improved relapse-free survival (*p* = 0.015). Exclusively nuclear immunoexpression of CXCR4 associated with better relapse-free survival (*p* = 0.032), but not after adjusting for vascular invasion. Patients with higher beta-catenin scores showed a tendency for poorer relapse-free survival (*p* = 0.056). MECA-79 immunoexpression was absent.

**Conclusions:**

The informative protein biomarkers (i.e., MIB-1, CXCL12, beta-catenin, and possibly CXCR4) may prove useful for risk-stratifying patients if validated in larger, multicentric and well-defined studies. Currently, classical histopathological features of testicular germ cell tumors remain key for relapse prediction.

## Background

Testicular germ cell tumors are among the most common solid neoplasms in young-adult males. Their overall good prognosis puts them on the top of most curable solid cancers, with survival rates above 85–90% [1]. Around 85% and 70–75% of stage I seminoma and non-seminoma patients, respectively, are cured with orchiectomy alone [2–5], meaning that a substantial amount of patients can be safely followed-up using surveillance, which is indeed being increasingly adopted [6]. However, still a subgroup of these patients relapses, most frequently during the first 2 years after initial diagnosis, and requires further treatments, which possibly lead to morbidity and mortality [7, 8]. For this reason, there is an urgent need for predictive biomarkers to assess the risk of stage I patients, and accurately discriminate those truly benefiting from adjuvant treatment to prevent relapses, from those that can safely be followed using surveillance to avoid early and late side effects of additional treatments on individuals most likely becoming long-term cancer survivors [9–11].

This risk stratification of stage I patients has so far relied on clinicopathological parameters, predominantly being vascular invasion and the amount of embryonal carcinoma (for non-seminomas) [12, 13] and size and *rete testis* invasion (for seminomas) [14]. With the exception of vascular invasion for non-seminomas, the prognostic power of the other biomarkers to guide treatment decisions is still under debate [14, 15]. Vascular invasion is the most discriminative biomarker so far, even in multivariable analyses [12, 13]. Recently, we confirmed the value of vascular invasion assessment in a surveillance cohort of stage I non-seminoma patients [16]. Moreover, we demonstrated that all patients depicting simultaneously lymph vessel and blood vessel invasion developed relapse; possibly, if validated, this should further identify high-risk patients. Overall, accurate pathological assessment is key since overdiagnosis (commonly observed in vascular invasion assessment by less experienced centers) may result in overtreatment [17, 18].

Other biomarkers have been studied for their prognostic/predictive value in testicular germ cell tumors, including MIB-1, CXCL12, CXCR4 and beta-catenin [19–22]. However, none has been introduced in the clinic yet, possibly since results among studies were not consistent or reflecting the variability among study designs. MECA-79 is another biomarker shown to be involved in antitumor responses in some malignancies, although so far not been explored in testicular germ cell tumors [23–25]. Also, for TEX19, a cancer testis antigen present in normal adult testis and involved in proliferation of several cancer types and of germ cells [26], its expression profile in testicular germ cell tumors has not been demonstrated yet. In this work we aim to assess the prognostic value of biomarkers related to proliferation (MIB-1, TEX19) and to the surrounding immune microenvironment (CXCL12, CXCR4, beta-catenin and MECA-79) in a cohort of stage I testicular germ cell tumor patients undergoing surveillance, including their impact in patient outcome, and to compare their performance to the classical histopathological variable vascular invasion.

## Methods

### Patients and samples

Patients undergoing orchiectomy and diagnosed with stage I testicular germ cell tumors (in the period between the years 1993–2018) were retrospectively queried from our dataset, which includes patients undergoing orchiectomy in several hospitals across the Netherlands. Pediatric (type I) and spermatocytic (type III) tumors were excluded (i.e. postpubertal, type II tumors, either seminomas or non-seminomas, were included). Only patients assigned to surveillance strategy (i.e. absence of any adjuvant treatment after the orchiectomy) were included in the study. Clinical files were reviewed by a clinician (WE) blinded to all the analyses performed. The following variables were collected: dates of birth, age and serum tumor biomarkers at diagnosis; histological subtype, laterality, size, presence of vascular invasion and *rete testis* invasion; treatment of relapses (surgery and/or chemotherapy); type and topography of relapses; and diagnosis, relapse, death and date of last follow-up. Relapse was categorized as “early” or “late” as indicated elsewhere, with a cutoff of 2 years after treatment [27]. Patients without the required clinical data were excluded.

Orchiectomy specimens were fixed overnight in 10% buffered formalin and representative sections were collected and paraffin embedded. All cases were histologically assessed by a germ cell tumor-dedicated Pathologist with decades of experience (JWO). Representative blocks (containing at least 1cm^2^ of tumor and interface with adjacent non-involved parenchyma) were selected for evaluation, of which serial 4-μm sections were cut and used for immunohistochemistry.

Use of patient samples was approved for research by the Medical Ethical Committee of the EMC (the Netherlands), permit no. 02.981. Samples were used according to the “Code for Proper Secondary Use of Human Tissue in The Netherlands” developed by the Dutch Federation of Medical Scientific Societies (FMWV, version, 2002; update 2011).

### Immunohistochemistry

Immunohistochemistry was performed with an automated, validated and accredited staining system (Ventana Benchmark ULTRA, Ventana Medical Systems, Tucsen, AZ, USA) using the optiview universal DAB detection Kit (cat.760–700, Ventana Medical Systems) or Ultraview detection kit (cat.760–500, Ventana Medical Systems). In brief, following deparaffinization and heat-induced antigen retrieval the tissue samples were incubated according to their optimized time with the antibody of interest (Supplementary Table 1). Incubation was followed by hematoxylin II counter stain for 12 min and then a blue colouring reagent for 8 min according to the manufactures instructions (Ventana Medical Systems, Tucsen, AZ, USA).

### Scoring of immunostainings

Scoring of immunostainings was performed by a germ cell tumor-dedicated investigator (J.L.), blinded to the clinical outcome/data of each case. Both the intensity (0, 1, 2 and 3, corresponding to absent, weak, intermediate and strong staining) and percentage of stained tumor cells were assessed independently, as done in previous studies assessing these markers. “weak”, “intermediate” and “strong” was defined as indicated in [28]. Overall assessment was reported for mixed tumors, since 17/23 consisted of tumors with > 90% embryonal carcinoma, with only small foci of cells from other distinct subtypes. The cellular localization of the staining was also annotated. For CXCL12, additionally to the staining in tumor cells, surrounding stromal/inflammatory cells were also scored by the same method. The final (relevant) scoring method of each immunostaining was then adjusted based on the overall staining patterns and to allow maximum comparability to previous studies (see below in the Results section).

### Statistical analysis

Data was tabulated using Microsoft Excel 2016 and analyzed using IBM SPSS Statistics version 24. Percentages were calculated based on the number of cases with available data and continuous variables were described as median plus interquartile range. Clinicopathological correlates were assessed for all samples and also within each histological subtype, and significant findings were reported. Associations between categorical variables were assessed using chi-square or Fisher exact test, as appropriate. Distribution of continuous variables (age and size) among groups was compared using the nonparametric Mann-Whitney U test. Survival analyses were computed with Kaplan-Meier estimator and log-rank test. Hazard ratios and respective 95% confidence intervals were estimated using Cox regression models. Statistical significance was set at *p* ≤ 0.05.

## Results

### Cohort characterization and impact of the various clinicopathological variables on patient outcome

A total of 70 patients met the including criteria of the study, 28 with seminoma and 42 with non-seminoma (detailed clinicopathological characterization of patients and tumor samples is depicted in Table 1, and separately for seminoma/non-seminoma in Table 2). Median follow-up time was 42 months. The median patient age at diagnosis was 32 years (interquartile range 28–39), 35 for seminoma and 26 for non-seminoma patient. Among non-seminomas, the most frequent histologies were mixed tumors (23/42, 54.8%, with embryonal carcinoma components being absent in only six of these), followed by pure embryonal carcinoma (16/42, 38.1%, due to special enrichment of the cohort for this histology). Overall, 28 patients (40.0%) developed relapse during the follow-up time, eight seminomas (28.6%) and 20 non-seminomas (47.6%). There were four late relapses (at 29, 27, 25 and just over 24 months), all corresponding to seminoma patients with recurrence of disease in retroperitoneal lymph-nodes, and one died of disease. There was a tendency for seminomas to be diagnosed in later age compared to non-seminomas (median age 35 vs. 30 years, *p* = 0.059). Non-seminomas depicted more frequently vascular invasion (*p* = 0.002), but there were no significant differences in tumor size or proportion of *rete testis* invasion.

**Table 1:** Clinicopathological features of stage I testicular germ cell tumor patients put on surveillance strategy

Variables	Cohort (*n* = 70)
Age [years (median, interquartile range)]	32 (28–39)
Laterality (n, %)	
Right	37/70 (52.9)
Left	33/70 (47.1)
Pre-operative serum AFP (n, %)	
Within normal range	49/70 (70.0)
Elevated	21/70 (30.0)
Pre-operative serum β-HCG (n, %)	
Within normal range	43/69 (62.3)
Elevated	26/69 (37.7)
Histologic subtypes (n, %)	
Pure seminoma	28/70 (40.0)
Pure embryonal carcinoma	16/70 (22.9)
Pure postpubertal-type yolk sac tumor	1/70 (1.4)
Pure postpubertal-type teratoma	2/70 (2.9)
Mixed tumor, with embryonal carcinoma component	17/70 (24.3)
Mixed tumor, without embryonal carcinoma component	6/70 (8.5)
Tumor size [cm (median, interquartile range)]	3.0 (2.0–4.3)
*Rete testis* invasion (n, %)	
Absent	50/66 (75.8)
Present	16/66 (24.2)
Vascular invasion (n, %)	
Absent	44/69 (63.8)
Present	25/69 (36.2)
Relapse (n, %)	
No	42/70 (60.0)
Yes	28/70 (40.0)
Type of relapse (n, %)	
Early	24/28 (85.7)
Late	4/28 (14.3)
Site of relapse (n, %)	
Only serum markers	7/28 (25.0)
Serum markers + PAoLN	6/28 (21.4)
Serum markers + Lung	2/28 (7.2)
Only PAoLN	9/28 (32.1)
Only Lung	3/28 (10.7)
PAoLN + Lung	1/28 (3.6)
Treatment performed for relapses (n, %)	
Refused therapy	1/28 (3.6)
Only chemotherapy (+/− radiotherapy)	23/28 (82.1)
Chemotherapy + RPLND	3/28 (10.7)
Chemotherapy + Lung resection	1/28 (3.6)
Vital status at last follow-up (n, %)	
A-NED	67/70 (95.7)
D-NED	1/70 (1.4)
DFD	2/70 (2.9)

*Abbreviations: AFP* alpha fetoprotein, *A-NED* alive with no evidence of disease, *β-HCG* human chorionic gonadotropin subunit beta, *DFD* died from disease, *D-NED* died with no evidence of disease, *PAoLN* para-aortic lymph-nodes, *RPLND* retroperitoneal lymph-node dissection

**Table 2:** Clinicopathological features of stage I seminoma/non-seminoma patients put on surveillance strategy

Variables	Seminomas (*n* = 28)	Non-seminomas (*n* = 42)	*p*-value
Age [years (median, interquartile range)]	35 (31–40)	30 (26–39)	0.059
Tumor size [cm (median, interquartile range)]	2.5 (1.3–4.1)	3.2 (2.0–5.0)	0.113
*Rete testis* invasion (n, %)			0.367
Absent	22/27 (81.5)	28/39 (71.8)	
Present	5/27 (18.5)	11/39 (28.2)	
Vascular invasion (n, %)			0.002
Absent	24/28 (85.7)	20/41 (48.8)	
Present	4/28 (14.3)	21/41 (51.2)	
Relapse (n, %)			0.111
No	20/28 (71.4)	22/42 (52.4)	
Yes	8/28 (28.6)	20/42 (47.6)	

**Fig. 1:**
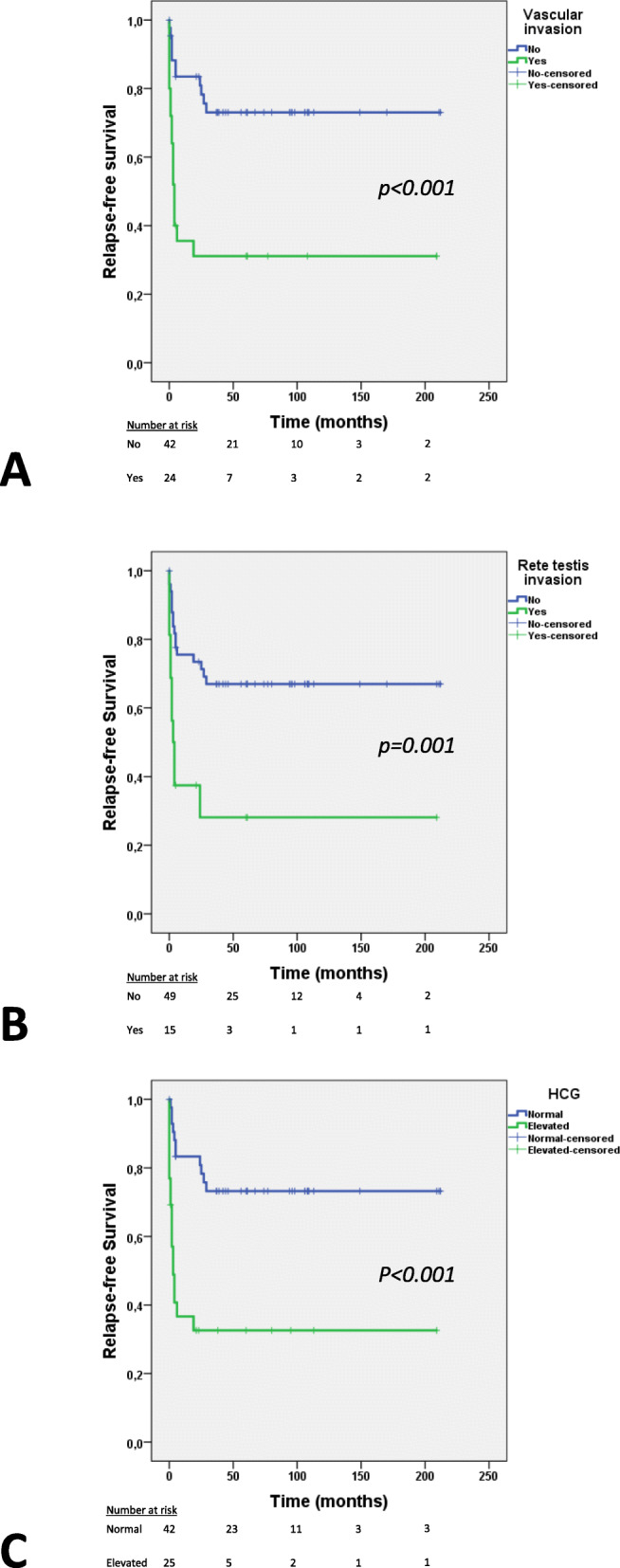
Kaplan-Meier curves regarding relapse-free survival in the stage I patient cohort on surveillance, according to clinicopathological features. **a** - vascular invasion; **b** - *rete testis* invasion; **c** - HCG elevation. Abbreviations: HCG – human chorionic gonadotropin

Overall, considering all patients (i.e. seminoma and non-seminoma), vascular invasion, *rete testis* invasion and elevation of human chorionic gonadotropin (HCG) were significantly associated with one another (vascular invasion vs. *rete testis* invasion: *p* < 0.001; vascular invasion vs. HCG elevation: *p* = 0.005), and all individually associated with disease relapse (*p* < 0.001, *p* = 0.009, *p* = 0.001, respectively). No significant associations were found between disease recurrence and histological subtype, alpha fetoprotein (AFP) elevation, tumor size or age at diagnosis. Regarding survival analysis, patients with vascular invasion experienced a worse relapse-free survival compared to those with no vascular invasion (hazard ratio 4.028, 95% confidence interval 1.868–8.682, *p* < 0.001), as did patients with *rete testis* invasion (hazard ratio 3.373, 95% confidence interval 1.545–7.364, *p* = 0.002) and elevation of HCG (hazard ratio 4.245, 95% confidence interval 1.964–9.178, p < 0.001) (Fig. 1a-c). The remaining variables did not show an impact of relapse-free survival. In multivariable analysis, vascular invasion showed an independent impact in relapse-free survival when adjusting for the effect of *rete testis* invasion (hazard ratio 2.864, 95% confidence interval 1.225–6.697, *p* = 0.015); when adjusting for the effect of HCG elevation, both variables remain significant (vascular invasion: hazard ratio 2.820, 95% confidence interval 1.257–6.328, *p* = 0.012; HCG: hazard ratio 3.025, 95% confidence interval 1.345–6.808, *p* = 0.007).

When stratifying the analysis according to histology, vascular invasion was found to be significantly associated with relapse in non-seminomas (*p* = 0.003), but not in seminoma patients (*p* = 0.555), while *rete testis* invasion and tumor size did not significantly associate with relapse in any of the groups, individually (*p* = 0.136 and *p* = 0.308 in seminomas; *p* = 0.060 and *p* = 0.477 in non-seminomas). Regarding all the non-seminoma patients, presence of embryonal carcinoma significantly associated with relapse as well (*p* = 0.022). In the next two sections the other findings based on immunohistochemistry will be presented, classified into two main categories, being related to proliferation (MIB-1 and TEX-19) and immune-micro-environment (CXCL12, CXCR4, beta-catenin, MECA-79*). q.*

### Biomarkers related to proliferation

#### MIB-1 scoring

Regarding MIB-1 scoring, intensity of staining (nuclear) was always high and did not vary among tumor samples, so we focused on the proportion of cells staining, like performed routinely for several tumor models (such as breast cancer [29]). The 40 and 70% cutoffs were assessed for comparability with previous studies on testicular germ cell tumors, and additionally other cutoffs were tested [21, 30, 31].

**Fig. 2:**
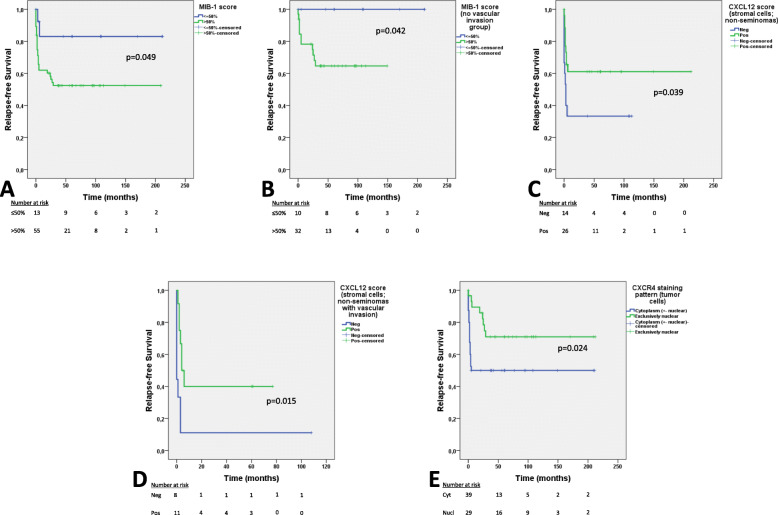
Kaplan-Meier curves regarding relapse-free survival in the stage I patient cohort on surveillance, according to immunoexpression of several markers. **a** - MIB-1 scoring using the 50% cutoff; **b** - MIB-1 scoring using the 50% cutoff (sub-analysis in patients with no vascular invasion only); **c** - CXCL12 positivity in surrounding stromal/inflammatory cells (sub-analysis of non-seminoma patients only); **d** - CXCL12 positivity in surrounding stromal/inflammatory cells (sub-analysis of non-seminoma patients displaying vascular invasion only); **e** - CXCR4 staining pattern in tumor cells

**Fig. 3:**
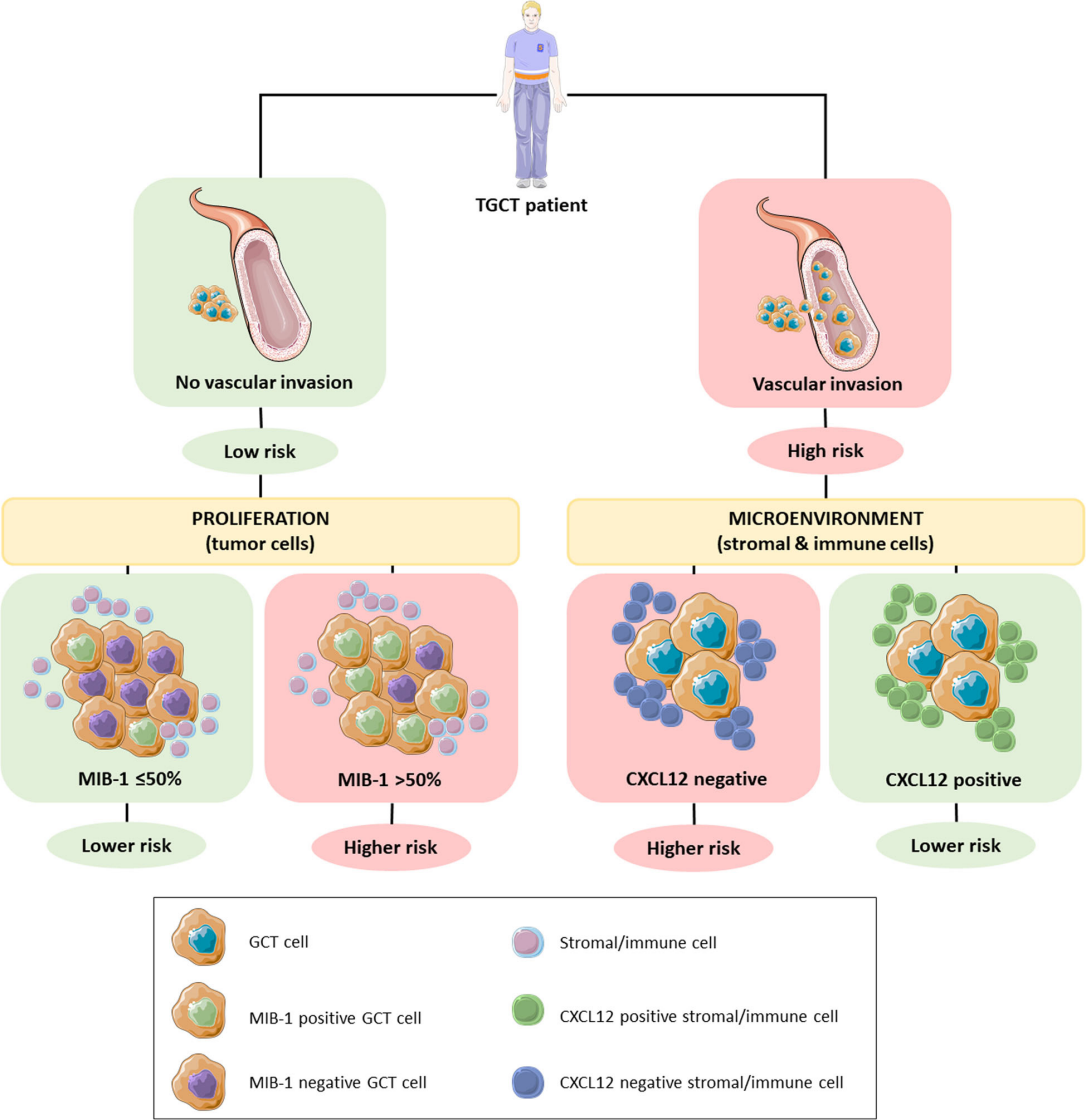
Biomarkers related to proliferation (MIB-1) and immune microenvironment (CXCL12) stratify risk groups in patients with and without vascular invasion. Abbreviations: GCT – germ cell tumor; TGCT – testicular germ cell tumor

Regarding the whole cohort of stage I patients, and considering referred cutoffs, there was no significant association between MIB-1 staining percentage and the event of relapse (*p* = 0.127). Although the relapse-free survival at 2 years was better for patients with ≤70% MIB-1 staining compared to those with > 70% staining (79% vs. 55%), the overall difference was not significant (*p* = 0.139). However, when considering the 50% cutoff, MIB-1 staining proportion significantly associated with relapse (*p* = 0.028, Table 3). Of the patients developing recurrence only 7% showed MIB-1 staining in ≤50% of the tumor cells. Patients with ≤50% MIB-1 staining experienced better relapse-free survival (*p* = 0.049, Fig. 2a). When adjusting for the effect of vascular invasion, MIB-1 staining loses its impact on relapse-free survival, and only vascular invasion remains significant (hazard ratio 3.628, 95% confidence interval 1.678–7.843, *p* = 0.001). However, when considering only patients without vascular invasion, MIB-1 staining further stratified the patients according to relapse-free survival, with those showing > 50% expression experiencing worse outcome (*p* = 0.042, Fig. 2b, Fig. 3). Besides the MIB-1 staining being significantly associated with vascular invasion in non-seminoma patients only (*p* = 0.014), it was not significantly associated with other pathological variables (histological type, vascular invasion, *rete testis* invasion, or tumor size).

**Table 3:** Immunoexpression of the several putative prognostic markers and associations with major clinicopathological variables

**Clinicopathological variables**	**MIB-1 ≤ 50%**	**MIB-1 > 50%**	***p*-value**
Relapse (% within relapse)			
No	12/42 (28.6)	30/42 (71.4)	0.028^a^
Yes	2/28 (7.1)	26/28 (92.9)	
Histology (% within histology)			
Seminoma	4/28 (14.3)	24/28 (85.7)	0.329
Non-seminoma	10/42 (23.8)	32/42 (76.2)	
Vascular invasion (% within vascular invasion)			
No	11/44 (25.0)	33/44 (75.0)	0.197
Yes	3/25 (12.0)	22/25 (88.0)	
Clinicopathological variables	**TEX19 negative**	**TEX19 positive**	***p*-value**
Relapse (% within relapse)			
No	34/42 (81.0)	8/42 (19.0)	0.552
Yes	21/28 (75.0)	7/28 (25.0)	
Histology (% within histology)			
Seminoma	27/28 (96.4)	1/28 (3.6)	0.003^a^
Non-seminoma	28/42 (66.7)	14/42 (33.3)	
Vascular invasion (% within vascular invasion)			
No	32/44 (72.7)	12/44 (27.3)	0.139
Yes	22/25 (88.0)	3/25 (12.0)	
Clinicopathological variables	**CXCL12 negative (tumor cells)**	**CXCL12 positive (tumor cells)**	***p*-value**
Relapse (% within relapse)			
No	27/42 (64.3)	15/42 (35.7)	0.533
Yes	20/28 (71.4)	8/28 (28.6)	
Histology (% within histology)			
Seminoma	27/28 (96.2)	1/28 (3.6)	< 0.001^a^
Non-seminoma	20/42 (47.6)	22/42 (52.4)	
Vascular invasion (% within vascular invasion)			
No	30/44 (68.2)	14/44 (31.8)	0.723
Yes	16/25 (64.0)	9/25 (36.0)	
Clinicopathological variables	**CXCR4 weak/moderate**	**CXCR4 strong**	***p*-value**
Relapse (% within relapse)			
No	8/42 (19.0)	34/42 (81.0)	0.506
Yes	3/28 (10.7)	25/28 (89.3)	
Histology (% within histology)			
Seminoma	8/28 (28.6)	20/28 (71.4)	0.021^a^
Non-seminoma	3/42 (7.1)	39/42 (92.9)	
Vascular invasion (% within vascular invasion)			
No	8/44 (18.2)	36/44 (81.8)	0.734
Yes	3/25 (12.0)	22/25 (88.0)	
Clinicopathological variables	**CXCR4 nuclear exclusive**	**CXCR4 non-nuclear exclusive**	***p*-value**
Relapse (% within relapse)			
No	22/42 (52.4)	20/42 (47.6)	0.049^a^
Yes	8/28 (28.6)	20/28 (71.4)	
Histology (% within histology)			
Seminoma	21/28 (75.0)	7/28 (25.0)	< 0.001^a^
Non-seminoma	9/42 (21.4)	33/42 (78.6)	
Vascular invasion (% within vascular invasion)			
No	26/44 (59.1)	18/44 (40.9)	< 0.001^a^
Yes	3/25 (12.0)	22/25 (88.0)	
Clinicopathological variables	**Beta catenin score ≤ 100**	**Beta catenin score > 100**	***p*-value**
Relapse (% within relapse)			
No	11/42 (26.2)	31/42 (73.8)	0.045^a^
Yes	2/28 (7.1)	26/28 (92.9)	
Histology (% within histology)			
Seminoma	11/28 (39.3)	17/28 (60.7)	< 0.001^a^
Non-seminoma	2/42 (4.8)	40/42 (95.2)	
Vascular invasion (% within vascular invasion)			
No	8/44 (18.2)	36/44 (81.8)	1.0
Yes	4/25 (16.0)	21/25 (84.0)	

^a^ Significant values

#### TEX19 scoring

TEX19 staining was found infrequently in our cohort (15/70, 21.4%), invariably corresponded to low intensity cytoplasmic staining in tumor cells and, in all cases but one, < 30% of cells showed expression (the exception being a single case of pure embryonal carcinoma with positivity in 80% of cells). In the absence of a reported cutoff we categorized cases as “positive” vs. “negative”. Nuclear staining was not seen on tumor cells.

Adjacent testicular parenchyma invariably showed strong cytoplasmic staining in cells in a basal position within the seminiferous tubule, corresponding to Sertoli cells. Small punctate foci of nuclear positivity were denoted in some scattered spermatogonial cells in normal testis (Supplementary Fig. 1).

Positivity was significantly more common in non-seminomas (*p* = 0.003, Table 3), with only one seminoma patient showing faint staining in tumor cells. There were no significant associations with the remaining pathological variables and expression did not associate significantly with disease relapse. Relapse-free survival at 5 months was of 72% for negative patients and of 53% for positive patients, but overall the impact on relapse-free survival did not reach statistical significance (Supplementary Fig. 2A).

### Biomarkers related to the immune microenvironment

#### CXCL12 scoring

When present, CXCL12 expression was always membrane/cytoplasmic (both in tumor and stromal/inflammatory cells), and was invariably strong in intensity, but occurring in a variable proportion of cells. According to the previous study, the 1 and 10% cutoffs were used to categorize the cases [21].

A total of 23 (32.9%) and 49 (70.0%) tumors showed expression of CXCL12 in tumor cells and surrounding stromal/inflammatory cells. Expression in tumor cells was significantly associated with non-seminoma histology (*p* < 0.001, Table 3), with only one seminoma case showing expression of the biomarker; no significant associations with other pathological variables were depicted, and expression (using both above mentioned cutoffs) did not associate with recurrence and did not show significant impact on relapse-free survival (*p* = 0.681, Supplementary Fig. 2B). The same was observed when stratifying the patients under investigation according to vascular invasion status. CXCL12 positivity in surrounding stromal/inflammatory cells (which was depicted in 22 seminoma and 27 non-seminoma patients) did not significantly associate with any histopathological variable; however, in non-seminoma patients specifically, it associated significantly with an improved relapse-free survival (*p* = 0.039, Fig. 2c). Moreover, this effect further stratified patients exhibiting vascular invasion, with those showing positivity for CXCL12 in immediate surrounding cells displaying better relapse-free survival (*p* = 0.015, Fig. 2d, Fig. 3).

#### CXCR4 scoring

CXCR4 expression in tumor cells was diffuse (90–100% of cells) in all but three (pure) seminomas. Intensity and location of staining was variable across tumor samples. Strong (vs. low/moderate) immunostaining intensity associated with non-seminoma histology (*p* = 0.021), but did not associate with the remaining pathological variables nor the event of relapse, and did not significantly influence relapse-free survival. Thirty-three tumors (47.1%) showed both cytoplasmic/membrane and nuclear expression of CXCR4, while 30 tumors (42.9%, 21 seminomas and nine non-seminomas) had exclusive nuclear staining. Seven tumors (all non-seminomas, of which five relapsed) showed exclusive expression on the cell membrane, without nuclear localization. Adjacent seminiferous tubules exhibited invariable nuclear expression of this marker. Exclusive nuclear immunoexpression (vs. other patterns) of CXCR4 significantly associated with seminoma histology (*p* < 0.001), absent vascular invasion (*p* < 0.001), absent *rete testis* invasion (*p* = 0.005) and absence of disease relapse (*p* = 0.049, Table 3). Patients with nuclear-only staining experienced a significantly improved relapse-free survival (hazard ratio 2.459, 95% confidence interval 1.079–5.607, *p* = 0.032, Fig. 2e). However, in multivariable analysis, only vascular invasion maintains an independent impact (hazard ratio 3.496, 95% confidence interval 1.503–8.134, *p* = 0.004).

#### Beta-catenin scoring

Regarding beta-catenin immunostaining patterns, membrane staining of tumor cells (and also of adjacent tubules, when present) was observed in all cases, with varying intensity and proportion. Hence, like reported before, we considered a combined multiplicative score of “intensity” x “percentage of stained cells”, and cases were then further grouped as “score ≤ 100” (hereon designated “low score”) and “score > 100” (hereon designated “high score”) [20].

Overall, a higher beta-catenin expression score significantly associated with non-seminoma histology (*p* < 0.001, Table 3) and with the event of relapse (*p* = 0.045), and did not associate significantly with the remaining pathological variables. Of all patients that developed relapse, only two (7%) had a low score, compared to 26 (93%) showing high score. In survival analysis, patients with higher score show a tendency for poorer relapse-free survival, although it did not achieve statistical significance (*p* = 0.056, Supplementary Fig. 2C).

#### MECA-79 scoring

MECA-79 was not found to be expressed in tumor cells of the included samples. Also, MECA-79-positive vessels were absent in all the samples tested.

**Fig. 4:**
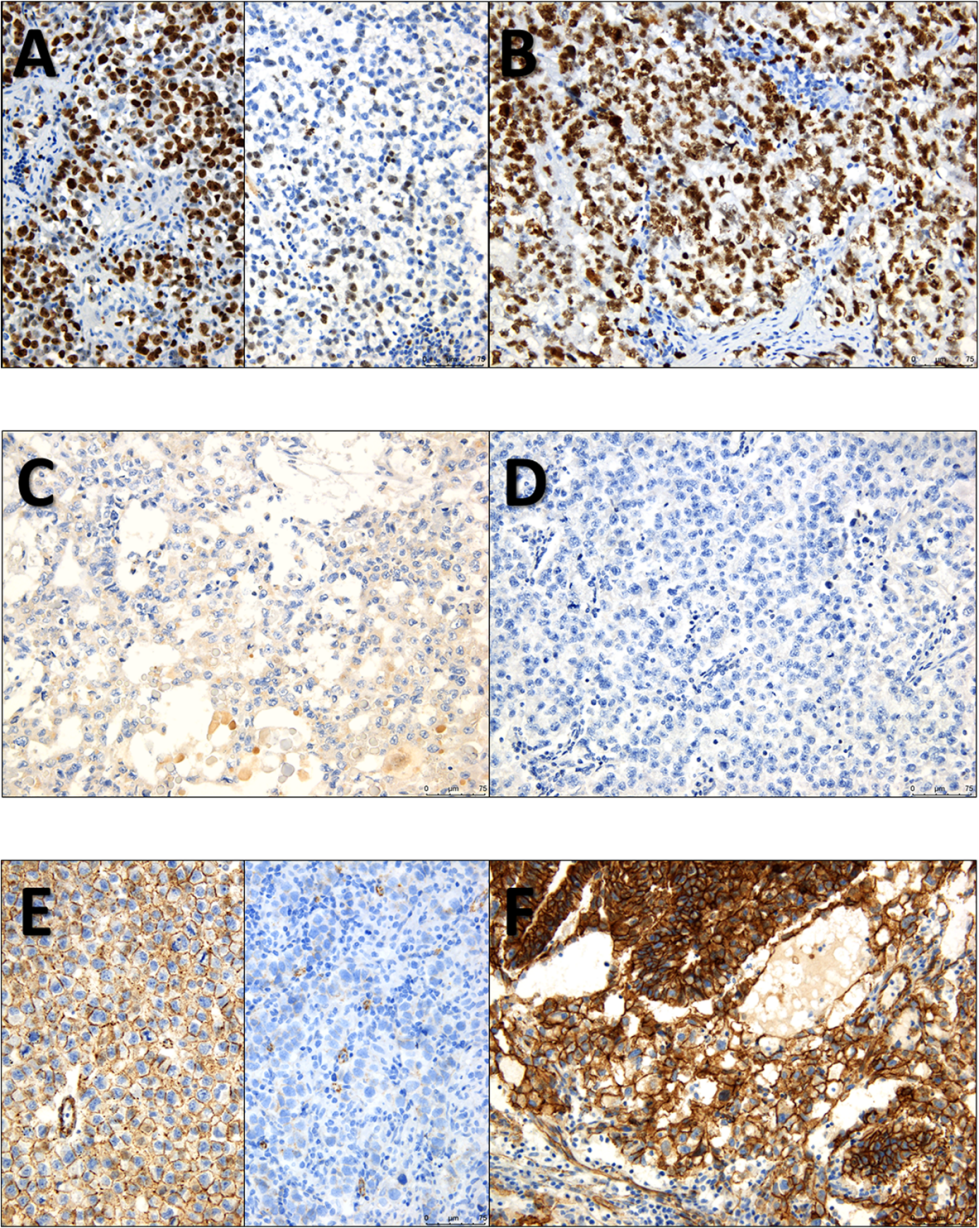
Representative examples of immunoexpression patterns of MIB-1, TEX19 and Beta-catenin. **a** – Two distinct pure seminoma cases, one with diffuse nuclear staining for MIB-1 (on the left) and another with < 50% positive tumor cells (on the right); **b** – A case of pure embryonal carcinoma with 100% positivity for MIB-1; **c** – Faint cytoplasmic positivity for TEX19 in a pure yolk sac tumor with hyaline globules; **d** – Complete absence of staining for TEX19 in a pure seminoma; **e** – Two distinct pure seminoma cases, one with diffuse, strong, membrane staining for Beta-catenin (on the left) and another with very faint and discontinuous membrane staining in 20% of tumor cells, rendering a combined score ≤ 100 (on the right). Notice the internal positive control (staining in capillary vessels within the tumor); **f** – Strong and diffuse membrane staining for Beta-catenin in a mixed tumor composed of yolk sac tumor, embryonal carcinoma and teratoma, rendering a combined score > 100

**Fig. 5:**
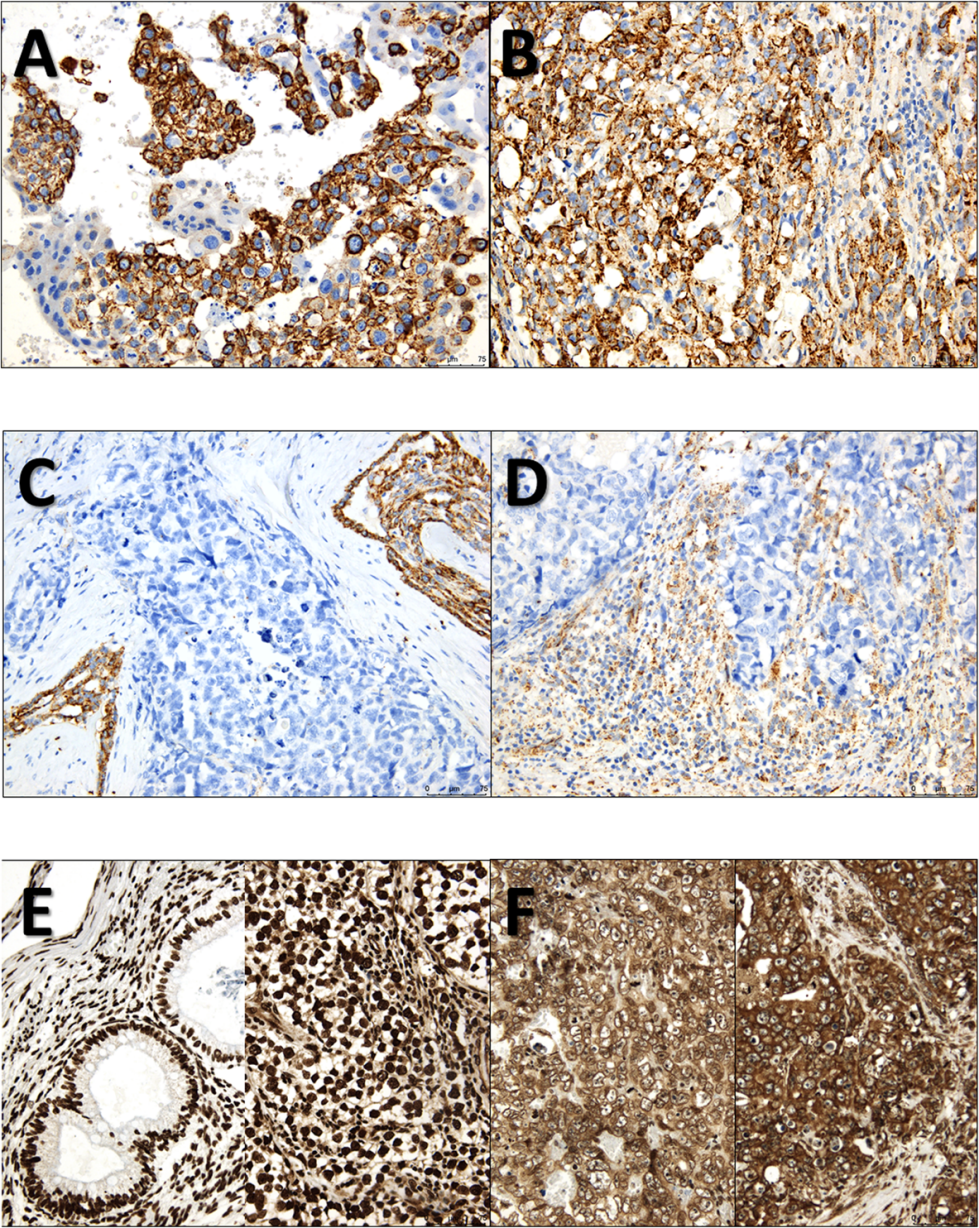
Illustrative examples of immunoexpression patterns of CXCL12 and CXCR4. **a** and **b** – Two cases with strong and diffuse membrane/cytoplasmic staining for CXCL12 in tumor cells, a choriocarcinoma component of a mixed tumor (**a**) and a yolk sac tumor component of a mixed tumor (**b**); **c** – A case of a pure embryonal carcinoma with absent immunoexpression of CXCL12 in tumor cells or in surrounding stromal/inflammatory cells. Notice the internal positive control in the *rete testis*; **d** – Notice the contrast to this case, another pure embryonal carcinoma with absent CXCL12 staining in tumor cells, but with abundant staining in surrounding stromal/immune cells in the tumor microenvironment, at the periphery and within the tumor nests; **e** – Two cases with exclusive strong nuclear immunoexpression of CXCR4, a pure teratoma (on the left) and a pure seminoma (on the right); **f** – Notice the contrast to these two cases of pure embryonal carcinomas, with a predominance of cytoplasmic staining for CXCR4. Rare nuclei exhibited CXCR4 staining in these cases

Representative pictures of the staining patterns of the various markers are depicted in Figs. 4 and 5 and in Supplementary Figs. 3 and 4. Storage time did not significantly influence the staining intensity/percentage across samples (Supplementary Fig. 5).

## Discussion

Testicular germ cell tumors are diagnostically challenging, especially because optimal pathological assessment of the primary tumor is key to determine the best therapeutic approach, predominantly in stage I disease [32]. In fact, accurate discrimination of high vs. low risk stage I patients is essential, in order to facilitate treatment decisions and avoid unnecessary side effects from chemotherapy and radiation as well as surgery. Assessment of this risk status has been largely dependent on histopathological variables, with vascular invasion being the most consistent prognostic tool for predicting disease relapse [22]. However, disagreements in scoring vascular invasion exist, more evident between peripheral and centralized centers with expertise on germ cell tumors, and these could result in over- and undertreatment [17, 18, 33]. Surveillance strategies are increasingly being employed in the approach to testicular germ cell tumor patients, given the outstanding cure rates of stage I disease with orchiectomy alone [6]. Still, there is a need of adjunctive biomarkers to complement the value of vascular invasion and other clinicopathological data.

Our work re-confirms the prognostic value of clinicopathological variables regarding disease relapse of stage I testicular germ cell tumor patients. Vascular invasion significantly associated with relapse in the overall cohort and significantly influenced relapse-free survival (including in multivariable analysis), and this impact was maintained when considering only non-seminoma patients, but not for seminoma patients, in line with previous studies [34, 35]. Presence of embryonal carcinoma is also associated with poorer relapse-free survival. This is in line with previous knowledge which unequivocally support the predictive value of vascular invasion assessment in non-seminoma patients [5, 12, 36–46] and further suggest amount of embryonal carcinoma as adjunctive in this context [5]. For seminoma, however, other variables are advanced to help in clinical decision-making: tumor size and *rete testis* invasion. In our series we did not however find these variables to be informative regarding disease recurrence; despite the limited number of patients included, this is in line with the systematic review of Boormans et al. [14] showing that the prognostic power of these features is still poor and cannot be reliably and blindly used to determine therapeutic action. Remarkably, elevation of HCG significantly associated with vascular invasion and it also significantly impacted relapse-free survival, even when adjusting for vascular invasion effect. It has been suggested that HCG elevations in testicular germ cell tumors (both seminomas and non-seminomas) positively regulate the vascular endothelial growth factor (VEGF), leading to vessel neoformation, which explains both the association with vascular invasion status and the poor prognostic value of this finding [47, 48].

The role of MIB-1 scoring as a prognostic marker in testicular germ cell tumors has been debatable, also because of different methodologies and cutoffs used in its assessment. While some studies demonstrated higher immunoexpression scores associating with poor prognosis (using the 40 and 70% cutoffs) [30, 31], more recent studies were not able to validate these cutoffs and reported this immunoexpression to be overall non-informative in a large cohort of stage I non-seminomas on surveillance, especially when adjusting to vascular invasion [21]. The distribution of MIB-1 staining proportions among these studies were also variable, possibly due to the different antibodies applied. In our work we could also not validate the 40 and 70% cutoffs. However, patients with > 50% scores experienced significantly poorer relapse-free survival, although the effect was again lost when adjusting for vascular invasion status. Importantly, when focusing solely on the subset of patients without vascular invasion, MIB scoring of more than 50% identified a group of patients with a worse clinical outcome. This finding, highlighted in Fig. 3, constitutes a novelty compared to previous studies mentioned above, and deserves validation in much larger cohorts to confirm its clinical usefulness.

TEX19 is a recently characterized player within the family of cancer testis antigens, which are normally expressed in the germ cell compartment of the normal testis, but also in a wide range of cancers, with current evidence pointing towards an oncogenic function of these proteins [49]. Planells-Palop et al. [26] revealed that TEX19 regulates proliferation and analysis of The Cancer Genome Atlas database shows it associates with distinct clinical outcomes in several tumor models. The authors evidenced cytoplasmic staining for the marker in basal cells within seminiferous tubules, which seemed to correspond to a subpopulation of Sertoli cells. This was in line with findings of Zhong et al. and did not support the role of this marker as a cancer testis antigen [50]. However, the former authors also evidence, by immunofluorescence, foci of nuclear expression of TEX19 in MAGE-A1-positive cells, corresponding to spermatogonia [26], a finding our study confirms by use of immunohistochemistry, as depicted in Supplementary Fig. 1. This supports the advanced role of TEX19 in germ cells, and not only in Sertoli cells, thereby fulfilling the classification of a cancer testis antigen. In addition, they demonstrate that this protein can accumulate both in the cytoplasm and nucleus of several cell lines, and describe staining in the nuclei of the germ cell tumor cell line NTera-2 (representative of a non-seminoma), contrasting to the absence of nuclear staining for this marker in our cohort of 70 stage I primary tumors. NTera-2 cells can easily develop signs of neural differentiation, which could affect the subcellular location of the protein. In our cohort we did find a weak cytoplasmic staining for TEX19 in a minority of primary tumors (i.e., 14 non-seminomas and one seminoma). The biological meaning of this finding is still unknown but indicates TEX19 may not behave solely as a cancer testis antigen. This biomarker was indeed advanced as contributing to tumor cells proliferation; in our series of stage I patients its expression did not, however, significantly affect clinical outcome, not did it impact disease-relapse.

The CXCL12/CXCR4 axis plays a role during male germ cell development, with primordial germ cells expressing CXCR4 and migrating towards locations with high CXCL12 content [51, 52]. In this line, there is rationale for exploring these markers in germ cell tumors, since they are developmental cancers [53, 54]. In two studies from the same group [21, 22] it was shown that a CXCL12 gradient was able to stimulate migration of germ cell tumor cells [22], and that non-seminoma stage I patients on surveillance with higher CXCL12 immunoexpression in tumor cells exhibited significantly better relapse-free survival, independently of vascular invasion status [21]. Authors hypothesized that the constant supply of CXCL12 might abrogate a gradient that is necessary to trigger migration. Our work, despite of using similar cutoff values, could not reproduce these findings, in line with recent observations by Fankhauser et al. [19]. However, all studies, including ours, demonstrate that CXCL12 is almost exclusively found in non-seminomas, and we confirmed this on an in silico analysis of The Cancer Genome Atlas (TCGA) database (Supplementary Fig. 6, *p* < 0.0001). So, we hypothesize that CXCR4/CXCL12 axis activation should be relevant for TGCT tumorigenesis. Indeed, Gilbert et al. demonstrated that CXCR4 expression led to activation of the PI3K-AKT and MEK-ERK pathways, constituting additional data showing the relevance of activated RAS pathway in TGCT tumorigenesis [22]. Given the known influence of the microenvironment in testicular germ cell tumors [28] we hypothesized that CXCL12 expression in stromal/inflammatory cells might also influence the final tendency for tumor cells to migrate and invade. Also, besides autocrine CXCL12 expression, peri-tumor immune cells may be inducing CXCL12 expression in the tumor cells they encase, in an attempt to prevent dissemination; this has not been explored before. Indeed, we found that non-seminoma patients with CXCL12 positivity in surrounding stromal/immune cells showed a significantly improved relapse-free survival, which was able to discriminate two risk groups within patients showing vascular invasion (*p* = 0.015). This finding has not been reported in previous works and deserves further validation. We believe that the high expression levels of CXCL12 in these cells immediately surrounding the tumor nests may also abrogate the necessary gradient for triggering migration of tumor cells towards other CXCL12-rich distant locations.

The receptor CXCR4 is present at the cell membrane, can be sequestered into the cytoplasm, but recent evidence also showed that it can translocate to the nucleus. Accurate detection of nuclear CXCR4 depends greatly on the antibody used and its ability to specifically target this relevant epitope [55]. Nuclear expression of CXCR4 has been shown to impact prognosis in several tumor models, associating both with poorer and improved outcome [56–58]. To the best of our knowledge, this has not been addressed in germ cell tumors. In our work, cases with exclusively CXCR4 nuclear immunoexpression were significantly more frequent in seminomas and significantly associated with better relapse-free survival. However, again, the effect was not maintained in multivariable analysis. We hypothesize that the absence of CXCR4 at the membrane renders the cell insensitive to any available CXCL12 gradient, and so these tumors may not migrate and invade.

Beta-catenin was demonstrated by Chovanec et al. as a poor prognostic factor in testicular germ cell tumors; the mechanism implied to be related to a suppressed immune microenvironment [20]. Using the same approach of the authors and applying a combined score of intensity and percentage of stained cells, we confirmed that the immunoexpression pattern is significantly lower in seminomas when compared to non-seminomas. Also, the authors did not find a significant impact of beta-catenin expression on relapse-free survival, including when stratifying the analysis for seminomas or non-seminomas only. However, they included both gonadal and extra-gonadal tumors, several disease stages and patients with metastatic disease receiving adjuvant treatment. In our more strict stage I surveillance setting we did find higher beta-catenin immunoexpression to be significantly associated with the event of relapse and a tendency for patients with higher expression to show overall poorer relapse-free survival (*p* = 0.045 and *p* = 0.056, respectively), which corroborates the immune-suppressed microenvironment initially described by the authors [20].

MECA-79 was documented to be ectopically expressed in tumor cells of gastric cancer patients (28% of the cases), which associated with poor prognostic features and poorer disease-specific survival [23]. Its expression has not been assessed in germ cell tumors to date. In our study, we found MECA-79 not to be expressed in tumor cells of both seminomas and non-seminomas. However, we specifically concentrated on the clinical setting of stage I disease on surveillance, with good prognosis, so it is possible that its expression is restricted to higher stage, advanced disease, like in the reported study on gastric cancer [23]. Moreover, MECA-79 has been used as a marker of the so-called high endothelial venules, which occur naturally in tertiary lymphoid organs and lymph nodes [59]. These specialized vessels are frequently found in solid tumors (demonstrated in colon, breast, lung and ovarian carcinoma and in malignant melanoma) and are thought to facilitate tumor infiltration by lymphocytes, contributing to better prognosis [60]. In malignant melanoma, for instance, a high density of such vessels was associated with tumor regression and good prognostic features [24]. Our work shows no presence of such MECA-79-positive vessels in all testicular germ cell tumor samples included (in the presence of staining of the positive control – human tonsil – depicted in Supplementary Fig. 4), indicating a limited role of this assessment as a predictive biomarker in this tumor model.

One of the limitations of our work is its retrospective nature and the relative small cohort size. However, the power is the true surveillance cohort of 70 stage I patients (partly already described in [16]), avoiding confounding factors related to any treatment except initial orchiectomy. Also, and because our cases derive from several institutions across the Netherlands, our findings do not reflect a selection bias due to inclusion of patients from a single center. Moreover, the classical prognostic histopathological variables already known to be informative in predicting relapse were re-confirmed in this work, validating our cohort.

## Conclusions

Overall, we believe that several protein biomarkers could bring additional value for the prediction of relapse in stage I testicular germ cell tumor patients, but further studies, large, multicentric and prospective, with defined methodology should be pursued before sound conclusions with clinical robustness can be made. For the time being, an accurate histopathological evaluation of the orchiectomy specimen by an experienced Pathologist and a correct clinical assessment of the patient are the most consistent tools for predicting patient outcome in the daily routine.

## Supplementary information

**Additional file 1: Supplementary Table 1.** Immunohistochemistry method details.

**Additional file 2: Supplementary Figure 1.** TEX19 expression in adjacent seminiferous tubules. TEX19 immunoexpression in normal testicular parenchyma. Notice the strong cytoplasmic staining restricted to the basal layer of the tubules, corresponding to Sertoli cells. Also notice the small punctate foci of nuclear staining in spermatogonial cells within the tubule (red arrows).

**Additional file 3: Supplementary Figure 2.** Kaplan-Meier curves regarding relapse-free survival in the stage I patient cohort on surveillance, according to immunoexpression of: TEX19 (A), CXCL12 positivity in tumor cells (B), and Beta-catenin combined score (C).

**Additional file 4: Supplementary Figure 3.** Representative examples of immunoexpression patterns of several markers in the adjacent testicular parenchyma. A – TEX19 cytoplasmic immunoexpression in tubules containing germ cell neoplasia in situ (GCNIS), localizing mainly to the basal layer in the position of Sertoli cells; B – TEX19 cytoplasmic immunoexpression in tubules containing GCNIS, and complete absence within tubules completely filled by GCNIS cells that contain no more Sertoli cells (intratubular seminoma); C – Strong and exclusively nuclear positivity for CXCR4 in seminiferous tubules adjacent to a seminoma. Staining of stromal/inflammatory cells is also depicted; D – Beta-catenin strong, diffuse, membrane/cytoplasmic immunoexpression in tubules containing multilayer GCNIS. Notice the contrast to lower intensity membrane staining in adjacent seminoma.

**Additional file 5: Supplementary Figure 4.** Representative examples of immunostaining patterns of MECA-79. A and B – Evidence of MECA-79-positive vessels within the positive control (human tonsil); C and D – Complete absence of MECA-79 immunoexpression, either in tumor cells (an example of an embryonal carcinoma is depicted) or in surrounding vessels.

**Additional file 6: Supplementary Figure 5.** Immunoexpression of the several markers in relation to time of storage of samples .

**Additional file 7: Supplementary Figure 6.** In silico analysis of CXCL12 expression within TCGA database for testicular germ cell tumors. mRNA expression levels from RNA-sequencing are plotted.

## Data Availability

All data generated or analysed during this study are included in this published article [and its supplementary information files].
