# Supplementary material for: Prediction of relapse in stage I testicular germ cell tumor patients on surveillance: investigation of biomarkers
Source: BMC Cancer. 2020 Aug 5;20:728. doi: 10.1186/s12885-020-07220-6 (PMC7405370; doi:10.1186/s12885-020-07220-6)
Supplement: Supplementary file 1 — Additional file 1: Supplementary Table 1. Immunohistochemistry method details. [file 12885_2020_7220_MOESM1_ESM.docx]

**Supplemental Digital Content 1. Immunohistochemistry method details**

| **Antibody** | **Type** | **Dilution** | **Company** | **Clone** | **Lot number** | **Procedure** | **Ab incubation at 37˚C** |
| --- | --- | --- | --- | --- | --- | --- | --- |
| MIB-1 | Anti-Rabbit | 2 µg/ml (RTU) | Ventana | *Ki-67* | 707946 | Ultraview CC1 36’ | 32 minutes |
| B-catenin | Anti-Mouse | 1:200 | BD | 14b-catenin | 3325857 | Optiview CC1 64’ | 60 minutes |
| CXCL12 | Anti-Mouse | 1:100 | R&D | 79018 | C0J0518081 | Optiview CC1 32' | 32 minutes |
| CXCR4 | Anti-Mouse | 1:1000 | R&D | 44716 | AVB1118071 | Optiview P1 4' | 32 minutes |
| TEX19 | Anti-Rabbit | 1:750 | Abcam | Polyclonal | GR3189227-3 | Optiview CC1 32' | 32 minutes |
| MECA79 | Anti-Rat | 1:50 | Santa Cruz | polyclonal | K1419 | Optiview CC1 32' | 32 minutes |
