# Supplementary figures and images for: Prediction of relapse in stage I testicular germ cell tumor patients on surveillance: investigation of biomarkers
Source: BMC Cancer. 2020 Aug 5;20:728. doi: 10.1186/s12885-020-07220-6 (PMC7405370; doi:10.1186/s12885-020-07220-6)

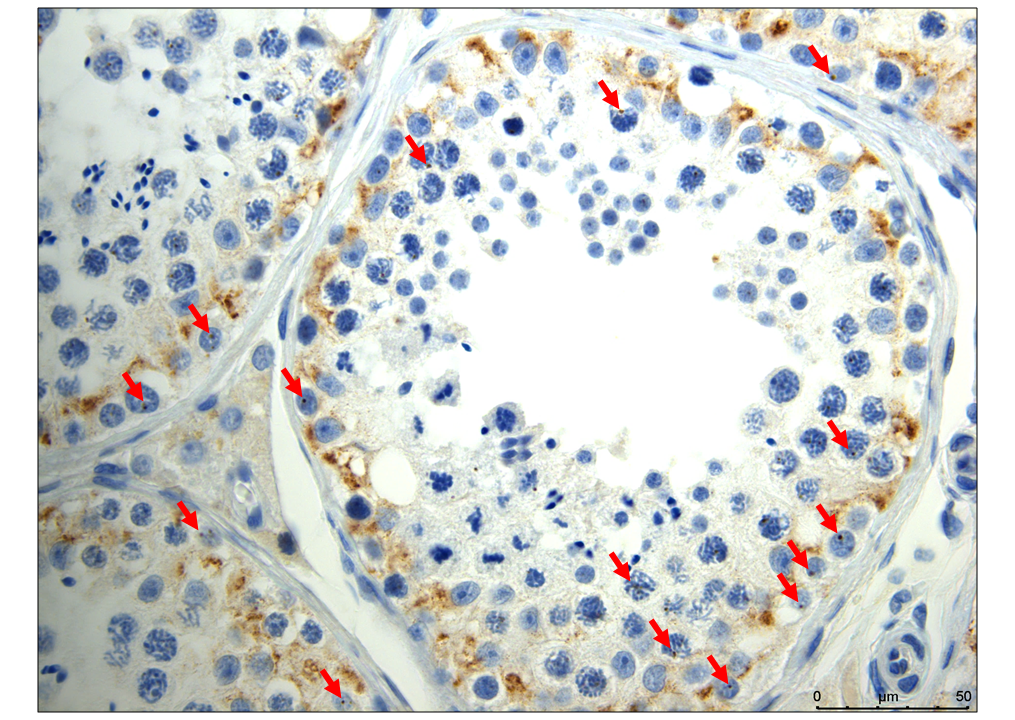

Supplement: Supplementary file 2 — Additional file 2: Supplementary Figure 1. TEX19 expression in adjacent seminiferous tubules. TEX19 immunoexpression in normal testicular parenchyma. Notice the strong cytoplasmic staining restricted to the basal layer of the tubules, corresponding to Sertoli cells. Also notice the small punctate foci of nuclear staining in spermatogonial cells within the tubule (red arrows). [file 12885_2020_7220_MOESM2_ESM.tif]

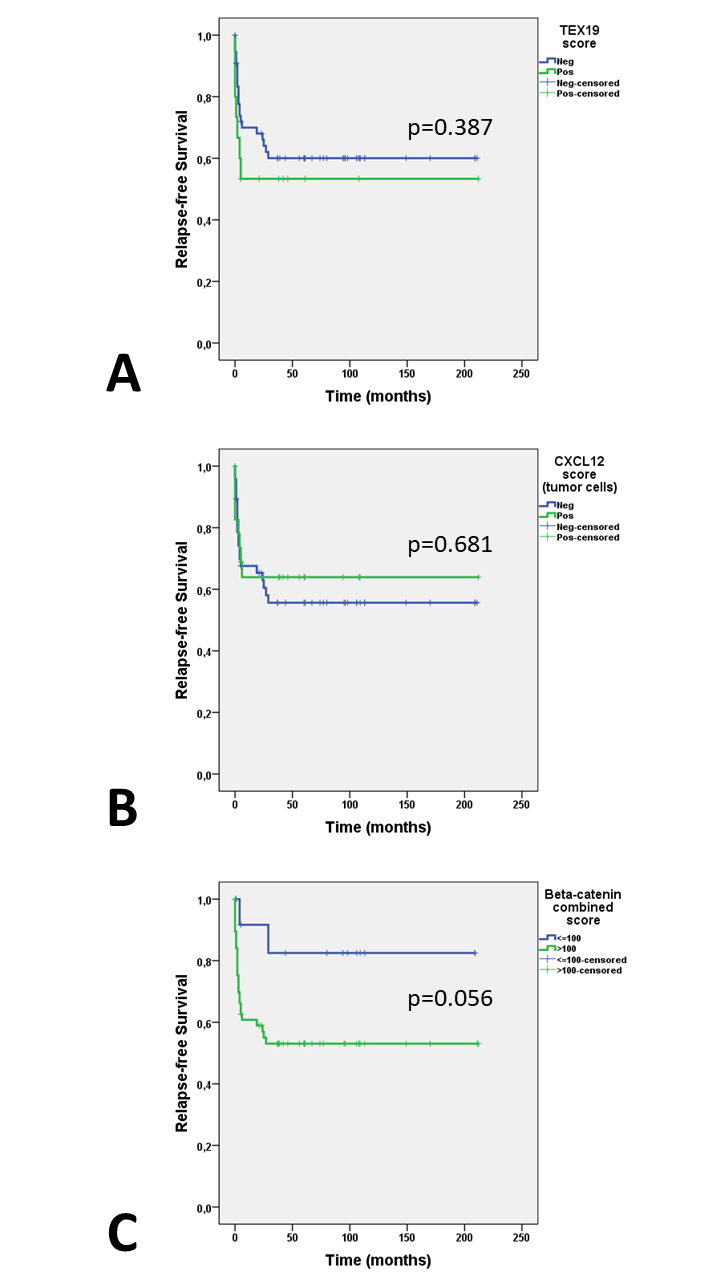

Supplement: Supplementary file 3 — Additional file 3: Supplementary Figure 2. Kaplan-Meier curves regarding relapse-free survival in the stage I patient cohort on surveillance, according to immunoexpression of: TEX19 (A), CXCL12 positivity in tumor cells (B), and Beta-catenin combined score (C). [file 12885_2020_7220_MOESM3_ESM.tif]

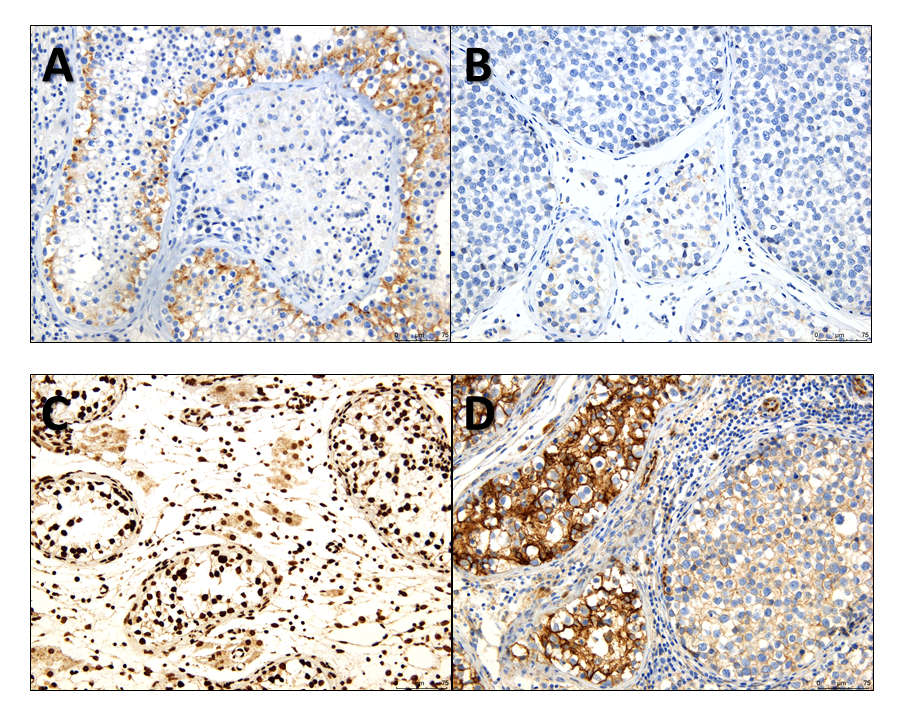

Supplement: Supplementary file 4 — Additional file 4: Supplementary Figure 3. Representative examples of immunoexpression patterns of several markers in the adjacent testicular parenchyma. A – TEX19 cytoplasmic immunoexpression in tubules containing germ cell neoplasia in situ (GCNIS), localizing mainly to the basal layer in the position of Sertoli cells; B – TEX19 cytoplasmic immunoexpression in tubules containing GCNIS, and complete absence within tubules completely filled by GCNIS cells that contain no more Sertoli cells (intratubular seminoma); C – Strong and exclusively nuclear positivity for CXCR4 in seminiferous tubules adjacent to a seminoma. Staining of stromal/inflammatory cells is also depicted; D – Beta-catenin strong, diffuse, membrane/cytoplasmic immunoexpression in tubules containing multilayer GCNIS. Notice the contrast to lower intensity membrane staining in adjacent seminoma. [file 12885_2020_7220_MOESM4_ESM.tif]

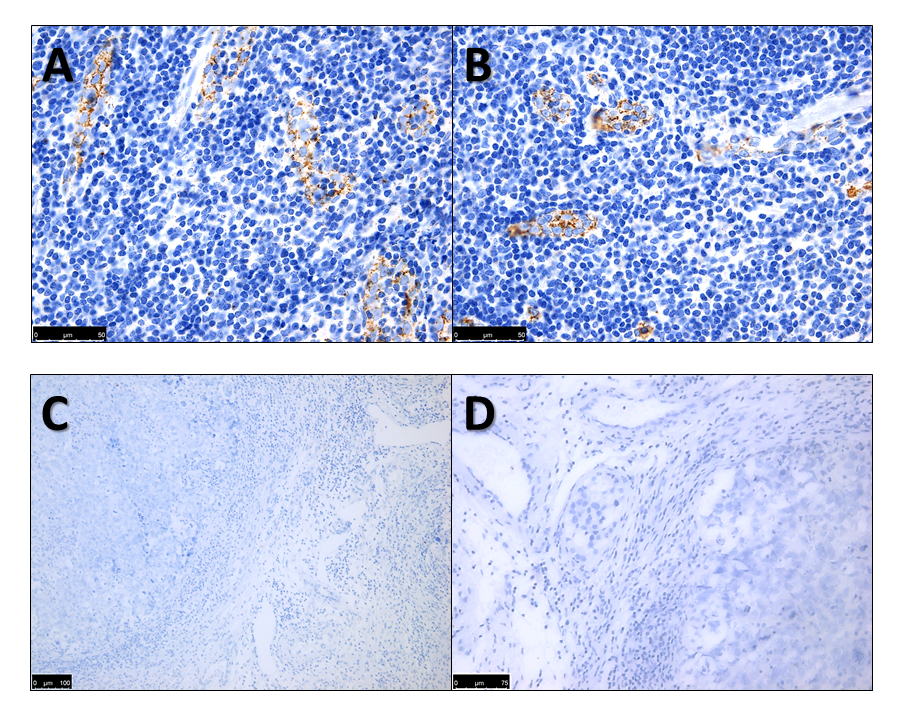

Supplement: Supplementary file 5 — Additional file 5: Supplementary Figure 4. Representative examples of immunostaining patterns of MECA-79. A and B – Evidence of MECA-79-positive vessels within the positive control (human tonsil); C and D – Complete absence of MECA-79 immunoexpression, either in tumor cells (an example of an embryonal carcinoma is depicted) or in surrounding vessels. [file 12885_2020_7220_MOESM5_ESM.tif]
